# Factors associated with the number of consultations per dietetic treatment: an observational study

**DOI:** 10.1186/1472-6963-12-317

**Published:** 2012-09-14

**Authors:** Jacqueline Tol, Ilse C Swinkels, Peter M Spreeuwenberg, Chantal J Leemrijse, Dinny H de Bakker, Cindy Veenhof

**Affiliations:** 1 NIVEL, Netherlands Institute for Health Services Research, Utrecht, The Netherlands; 2 Scientific Centre for Transformation in Care and Welfare (TRANZO), Tilburg University, Tilburg, The Netherlands

## Abstract

**Background:**

Greater understanding of the variance in the number of consultations per dietetic treatment will increase the transparency of dietetic healthcare. Substantial inter-practitioner variation may suggest a potential to increase efficiency and improve quality. It is not known whether inter-practitioner variation also exists in the field of dietetics. Therefore, the aims of this study are to examine inter-practitioner variation in the number of consultations per treatment and the case-mix factors that explain this variation.

**Methods:**

For this observational study, data were used from the National Information Service for Allied Health Care (LiPZ). LiPZ is a Dutch registration network of allied health care professionals, including dietitians working in primary healthcare. Data were used from 6,496 patients who underwent dietetic treatment between 2006 and 2009, treated by 27 dietitians working in solo practices located throughout the Netherlands. Data collection was based on the long-term computerized registration of healthcare-related information on patients, reimbursement, treatment and health problems, using a regular software program for reimbursement. Poisson multilevel regression analyses were used to model the number of consultations and to account for the clustered structure of the data.

**Results:**

After adjusting for case-mix, seven percent of the total variation in consultation sessions was due to dietitians. The mean number of consultations per treatment was 4.9 and ranged from 2.3–10.1 between dietitians. Demographic characteristics, patients’ initiative and patients’ health problems explained 28% of the inter-practitioner variation. Certain groups of patients used significantly more dietetic healthcare compared to others, i.e. older patients, females, the native Dutch, patients with a history of dietetic healthcare, patients who started the treatment on their own initiative, patients with multiple diagnoses, overweight, or binge eating disorder.

**Conclusions:**

Considerable variation in number of consultations per dietetic treatment is due to dietitians. Some of this inter-practitioner variation was reduced after adjusting for case-mix. Further research is necessary to study the relation between inter-practitioner variation and the effectiveness and quality of dietetic treatment.

## Background

Nowadays, dietitians are working in many countries throughout the world. Although countries can differ in the proportion of dietitians in the population and in professional qualifications, they all share the aim that dietitians should provide effective treatments based on scientific evidence about appropriate nutritional care in health and disease [1]. Several clinical studies have shown that treatment by a dietitian can be effective [2-6]. However, these studies were performed in small settings with specific groups of patients. To evaluate the effectiveness of dietetic treatment, large monitoring studies in public health and primary care settings are needed [7]. Transparency to all aspects of dietetic treatment is a prerequisite for the success of studies on effectiveness. Transparency can be increased, for example by focusing on inter-practitioner variation in dietetic treatment. Inter-practitioner variation in primary health care settings has been studied for several years. Many studies have shown that substantial inter-practitioner variation may suggest a potential to increase efficiency and improve quality [8-11]. It is not known whether inter-practitioner variation also exists in the field of dietetics. Multiple aspects of dietetic treatment can be used to examine inter-practitioner variability, such as the number of consultations per treatment.

In general, variation in the number of consultations per treatment can occur on different levels, e.g. that of the organization, therapists and patients. First, on the level of the organization variation may be due to the organization’s treatment policy, the work environment, or cost and reimbursement issues [12]. Second, variation due to therapists may be attributable to differences between dietitians in professional experience, communication skills, expertise in effecting behavioral change, and beliefs about dietetic counseling [13-16]. These differences can influence the therapeutic decision-making process and the ability to teach patients new knowledge, skills and perception [17]. Third, variation on patient level may be due to demographics, health status [18,19] or behavioral issues, including locus of control, socio-environmental factors, intentions and motivation [20]. Patients with complex health problems are probably in need of more dietetic healthcare. Consequently, they are more likely to have a history of dietetics. Dutch dietitians expected patients with multiple diagnoses, psychological problems, or communication problems to be associated with a higher consultation rate [21]. However, case-mix effects have not been studied in relation to the number of consultations per dietetic treatment.

More knowledge about variation in consultation sessions might help to eliminate unwanted variation (e.g. variation not explained by disease, patient preference or evidence based medicine) in treatment. This could contribute towards improving the quality of dietetics and reducing unnecessary healthcare costs. Accordingly, the aims of this quantitative study are: 1) to examine the inter-practitioner variation in the number of consultations per treatment; 2) to determine the association between the number of consultations per treatment and case-mix variables.

## Methods

### Study design

For this longitudinal observational study, data were used from the National Information Service for Allied Health Care (LiPZ). LiPZ is a Dutch national registration network for allied healthcare professionals [22], including registered dietitians (RDs) working in primary healthcare. Relevant information on the organization of dietetics in the Netherlands is provided in Additional file 1.

### LiPZ participants

Recruitment started in 2005 by contacting 42 RDs who had indicated an interest in participating in LiPZ according to a previous questionnaire-based study of a representative sample of 500 Dutch primary health care working dietitians. Additionally, an announcement about participation in LiPZ was placed on the website of the software program (Evry) frequently used by dietetic practices. RDs were included if they recorded patient, treatment, and reimbursement information (see Additional file 1) in the software program Evry. No exclusion criteria were applied in order to participate. A total of 27 RDs working in solo practices were enrolled. This sample size accounts for 3% of the total population of Dutch dietitians working in private practices and is sufficient in order to be representative for practice region, and level of urbanicity. When drop-outs occurred, it was intended to keep the network as representative as possible. Therefore, new dietitians were invited and if possible selection was based on practice region and level of urbanicity. The reason for dropping out was often because participating was too time-consuming. In return for participating, the RDs received among others a financial compensation and points for accreditation in the quality register.

### LiPZ data collection

Information about the dietitians’ demographics was collected by a self-reported questionnaire at the time of enrollment. Patient data collection was based on extractions from electronic medical records. The records consisted of long-term computerized registration of healthcare-related information. Dietitians recorded all data needed for reimbursement routinely, e.g. patient’s age and gender and dates of consultation. Furthermore, a special LiPZ-module was installed in the software program to register supplementary information on patient’s treatment, for example on educational level, history in dietetics, health problems and initiative of treatment. Information on initiative of treatment was collected as dietitians were accessible only via referral by a physician (see Additional file 1). However, it was possible that patients could have initiated treatment by, for example asking for a referral to a dietitian. Data were submitted by the participants on a monthly basis. After submitting new data, standardized quality control checks on missing or inconsistent data were carried out by research assistants. Consequently, the participants received an overview of the missing or inconsistent data and were asked to complete or adjust data accordingly. Ethical approval for this study was not required, since the patients received customary care without experimental interventions. Nevertheless, the Dutch Data Protection Authority was notified. In addition, pursuant to the Personal Data Protection Act data were collected anonymously; patients were informed about the LiPZ study by posters and leaflets in the practice waiting rooms, and they could opt not to participate in the study.

### Study sample and outcome

The study sample was based on data from LiPZ. Between 1 January 2006 and 1 January 2010, 8,320 new patients within 27 solo practices completed dietetic treatment. The data were collected at the level of a consultation and consultations were clustered within one treatment. One treatment includes all patients’ consultations for the same health problem. For this study, patient records with missing values were excluded (n=1,824). The total number of consultations (face-to-face contact) per dietetic treatment was used as the outcome of this study. Table 1 explains the measurement of the case-mix variables used in this study.

**Table 1:** Measurement of case-mix variables used in this study

**Variables**	**Measurement**	**Used in analyses as**
**Patients’ demographics (model 1)**		
Gender		Categorical: Male ^a^; Female
Age	Date of birth	Continuous: years of age at start of the treatment.
Ethnicity	Patient’s origin	Categorical: Immigrant ^a^, from a non-western country, i.e. Turkey, Africa, Latin-America and Asia; Native Dutch, including western immigrants originally from Europe, North-America, Oceania, Indonesia, Japan.
Educational level	Highest level of education	Categorical: Low (Primary school) ^a^; Medium (Secondary- or higher education); High (University); Other (not specified, e.g. in children).
Urbanicity	Zip-code of the address	Categorical: High (≥ 1500 addresses per km^2^); Medium (1000–1499 addresses per km^2^); Low (< 999 addresses per km^2^).
History in Dietetics	The patient had previous dietetic health care in the past 5 years.	Categorical: No ^a^; Yes.
**Patients’ initiative (model 2)**		
Initiative treatment	The patient was referred to the dietitian initiated by the referrer or the patient.	Categorical: Referrer ^a^; Patient.
**Patients’ health (model 3)**		
Communication problem	According to the dietitian	Categorical: No ^a^, Yes.
Psychiatric problem	According to the dietitian	Categorical: No ^a^, Yes.
Intellectual disability problem	According to the dietitian	Categorical: No ^a^, Yes.
Multiple diagnoses	Number of diagnoses per patient (max. 4)	Categorical: Single diagnosis ^a^, multiple diagnosis
Overweight	Type of diagnosis according to the dietitian	Categorical: No ^a^; Yes.
Underweight/unwanted weight loss	Type of diagnosis according to the dietitian	Categorical: No ^a^; Yes.
Hypercholesterolemia	Type of diagnosis according to the dietitian	Categorical: No ^a^; Yes.
Diabetes	Type of diagnosis according to the dietitian	Categorical: No ^a^; Yes.
Food intolerance	Type of diagnosis according to the dietitian	Categorical: No ^a^; Yes.
Irritable Bowel Syndrome	Type of diagnosis according to the dietitian	Categorical: No ^a^; Yes.
Binge eating disorder	Type of diagnosis according to the dietitian	Categorical: No ^a^; Yes.
Overweight & Diabetes;	Type of diagnosis according to the dietitian	Categorical: No ^a^; Yes.
Overweight & Hypertension	Type of diagnosis according to the dietitian	Categorical: No ^a^; Yes.
Overweight & Hypercholesterolemia;	Type of diagnosis according to the dietitian	Categorical: No ^a^; Yes.
Overweight & Diabetes & Hypercholesterolemia;	Type of diagnosis according to the dietitian	Categorical: No ^a^; Yes.
Overweight & Diabetes & Hypertension;	Type of diagnosis according to the dietitian	Categorical: No ^a^; Yes.
Overweight & Binge eating disorder;	Type of diagnosis according to the dietitian	Categorical: No ^a^; Yes.
Overweight & Irritable Bowel Syndrome	Type of diagnosis according to the dietitian	Categorical: No ^a^; Yes.
**Therapists’ demographics (model 4)**		
Gender		Categorical: Male ^a^; Female
Age	Date of birth	Continuous: years of age at start of the study.

^a^ These categories were used as reference group.

### Statistical analyses

The characteristics of the patients and the dietitians were analyzed using descriptive statistics in STATA (version 11, 2009, STATACorp, College Station Texas). Categorical variables were presented as percentages, and continuous variables were presented as mean values with standard deviations or median values with interquartile range (IQR) for non-normally distributed variables.

The data were analyzed using multilevel poisson regression analyses in MLwiN (Version 2.15, 2009, Centre for Multilevel Modeling, University of Bristol) [23,24]. Multilevel analyses were used to take into account the structure of the data: patients were nested within dietitians. The model therefore consists of two levels: the patient level (level 1) and the dietitian level (level 2). Because the outcome variable was a count variable, poisson regression was performed [25]. Several models were developed to fit the data, namely: model 0) the intercept-only model; model 1) patients’ demographics; model 2) which included the variables of model 1 and patients’ initiative; model 3) which included variables of model 2 variables on patients’ health; model 4) which included the variables of model 3 and therapists’ demographics. The analyses were carried out in several steps. First, the variance partition coefficient (VPC) was calculated for all models to express the inter-practitioner variation [24]. The VPC on dietitian level indicates the influence of the dietitians on consultation sessions that cannot be explained by the model parameters. Secondly, the proportional change in variance estimates of the different models was calculated. This indicates the part that case-mix factors explain concerning the total inter-practitioner variation [24]. The variance estimate is similar to the R^2^ in traditional regression, except that it focuses on specific level variance and not on total variance. Finally, regression coefficients, standard errors and p-values were calculated for all variables in model 4, to examine the association between the number of consultations per treatment and case-mix factors. A P-value of ≤ 0.05 was considered to be statistically significant.

## Results

Almost all dietitians in this study were female (n=25). The majority of the patients were female, native Dutch, had second-level education and lived in a highly urbanized area. Descriptive characteristics of the therapists and patients are presented in Table 2. The raw number of consultations per treatment varied between patients from 1–56 consultations, with a median of 4 and IQR of 2–7 consultations per treatment.

**Table 2:** Descriptive statistics and poison multilevel regression-analysis of case-mix variables on consultation sessions (n=6,496)

	**Descriptive**	**Poisson regression**		
	**Mean ± sd **^**a **^**Percentages**	**Regression coefficient**	**SE **^**b**^	**P-value**
**Intercept**		−0.72	0.07	
**Patients’ demographics**				
Female (reference male)	65.2%	0.09	0.02	<0.001
Age (years)	44.8 ± 19.1	−0.00	0.00	0.002
Native ethnicity (reference immigrants)	88.5%	0.11	0.04	0.003
Educational level:				
Low (reference)	31.3%			
Medium	42.2%	−0.08	0.02	<0.001
High	23.7%	−0.08	0.03	0.002
Not specified	2.9%	−0.05	0.06	0.422
Urbanicity:				
Low	34.8%	0.02	0.03	0.476
Medium	24.9%	−0.03	0.03	0.284
Strong (reference)	39.9%			
History in dietetics (reference no history)	22.3%	0.14	0.02	<0.001
**Patients’ initiative**				
Start treatment initiated by the patient (reference by referrers initiative)	14.5%	0.13	0.03	0.024
**Patients’ health**				
Communication problem (reference no communication problems)	3.9%	−0.02	0.05	0.717
Psychiatric problem (reference no psychiatric problems)	9.2%	0.12	0.03	<0.001
Intellectual disability problem (reference no problems with respect to intellectual disability)	2.0%	0.06	0.07	0.385
Multiple diagnoses (reference single diagnosis)	52.0%	0.36	0.05	<0.001
Overweight ^c^	30.6%	0.50	0.04	<0.001
Underweight, unwanted weight loss ^c^	3.0%	0.06	0.07	0.434
Hypercholesterolemia ^c^	2.8%	−0.33	0.09	<0.001
Diabetes Mellitus ^c^	2.5%	−0.04	0.09	0.672
Irritable Bowel Syndrome ^c^	1.6%	−0.15	0.10	0.132
Food intolerance ^c^	0.6%	−0.31	0.16	0.054
Binge eating disorder ^c^	0.5%	0.32	0.13	0.016
Overweight & Diabetes ^c^	8.0%	0.07	0.04	0.052
Overweight & Hypercholesterolemia ^c^	4.8%	−0.00	0.05	0.935
Overweight & Hypertension ^c^	4.3%	0.14	0.05	0.003
Overweight & Diabetes & Hypercholesterolemia ^c^	4.3%	0.04	0.05	0.444
Overweight & Diabetes & Hypertension ^c^	3.2%	0.14	0.06	0.015
Overweight & Binge eating disorder ^c^	1.6%	0.14	0.07	0.043
Overweight & Irritable Bowel Syndrome ^c^	1.3%	0.00	0.07	0.952
**Therapists’ demographics**				
Age (years)	46.0 ± 6.1	−0.01	0.01	0.539
Female (male)	93.6%	0.60	0.30	0.048

^a^*sd* standard deviation.

^b^*SE* standard error.

^c^ The reference group is having another diagnosis or a combination of diagnoses.

### Inter-practitioner variation in the number of consultations per treatment

Without correcting for case-mix factors, the VPC on dietitian level was 10.4% (intercept only model). The inter-practitioner variation decreased to 7.1% after including case-mix factors into the model, i.e. demographics, patients’ initiative and patients’ health-related variables. This indicates the influence of dietitians on consultation sessions that cannot be explained by the model parameters (see Table 3). Adjusted for these variables, the mean number of consultations was 4.9 and varied between dietitians with a 95% coverage interval from 2.3–10.1 consultations per treatment.

**Table 3:** Explaining inter-practitioner variation in the number of consultations per dietetic treatment

	**Intercept only model**	**Model 1 **^**a**^	**Model 2 **^**b**^	**Model 3 **^**c**^	**Model 4 **^**d**^
**Variance estimate**	0.1909	0.1694	0.1621	0.1573	0.1378
**Proportional reduction in variance estimates compared to the intercept only model**		11.3%	15.1%	17.6%	27.8%
**Variance partitioning coefficient**					
Therapist level	10.4%	9.0%	8.6%	8.2%	7.1%

^a^ Model 1 included patients’ demographic variables.

^b^ Model 2 included variables of model 1 + patients’ initiative.

^c^ Model 3 included variables of model 2 + patients’ health-related variables.

^d^ Model 4 included variables of model 3 + therapists’ demographic variables.

The case-mix factors of this study explained 27.8% of the inter-practitioner variation. Most of this variation (11.3%) was explained by patients’ demographics (model 1). The variation between dietitians’ consultations was further explained by adding patients’ initiative (3.8%), patients’ health-related variables (2.5%), and therapist demographics (10.2%) to the model.

### Case-mix associated with the number of consultations per treatment

The association between the case-mix variables of model 4 and consultation sessions is presented in Table 2. Patient characteristics that were significantly associated with a higher number of consultations per treatment were: females, natives, patients who have had dietetic health care in the past, and patients who started the treatment by own initiative. Health related variables that were associated with a higher number of consultations per treatment were patients with: psychiatric problems, overweight, binge eating disorder, multiple diagnoses, overweight in combination with diabetes and hypertension, overweight and hypertension, overweight and binge eating disorder. Patients with hypercholesterolemia were significantly associated with having less consultations per treatment compared to patients with a different diagnosis. Other patient characteristics that were significantly associated with a lower number of consultations were older patients, and patients with a medium or high educational level compared to patients with a low educational level.

## Discussion

The results show that considerable variation in number of consultations per treatment is due to dietitians. Seven percent of the total variance was concentrated at dietitian level. Compared to some other studies examining inter-practitioner variance, this percentage seems rather high [8,18,26-28]. In absolute terms, the mean number of consultations varied widely between dietitians, from 2.3 to 10.1 consultations per treatment. The inter-practitioner variance was partly (28%) explained by demographic characteristics, patients’ initiative and patients’ health problems. This is relatively high compared to studies in other professions [18,29]. Therefore, when studying inter-practitioner variation on dietitian level it is important to adjust for case-mix factors. This is especially the case for demographic characteristics as the patient’s health problems only explained 2.5% of the variation between dietitians in the number of consultations per treatment. The results from this study indicate that similar patients receive different dietetic care, which might raise questions for future studies. For example, whether there is under or over-use of dietetic care resources and unnecessary health care costs. Therefore, future studies should focus on examining other kinds of inter-practitioner variance and whether this variance is appropriate or not. Appropriate variation might be related to the clinical health status of the patient [19]. Inappropriate variation might be due to non-medical factors, such as differences in counseling styles [15] or workload as small list sizes can be associated with high consultation rates [30]. Furthermore, high levels of inter-practitioner variation might raise questions about the quality of care, although the level of variation is not directly linked to the quality of care. Therefore, results of this particular study cannot be used to draw conclusions on the quality of dietetic treatment. Further research on consultation rate and the effectiveness and quality of dietetic treatment is necessary.

Demographic characteristics of the patients were associated with the number of consultations sessions. These results were in accordance with studies in other healthcare professions [18,31,32]. However, the positive association between patients’ age and a lower number of consultations per treatment was not in accordance with other studies [18,29]. Possibly, the expectations of elderly patients in terms of aims to achieve or personal wishes are lower compared to younger patients. Furthermore, immigrants were associated with having fewer consultations per treatment compared to the native Dutch population. This was not in accordance with the expectation of Dutch dietitians [21]. Ethnic background in itself cannot explain differences in healthcare use. However, language and cultural differences may be the underlying issue accounting for difference in healthcare utilization [33]. For example, if a dietitian is not aware of the cultural differences around food, he or she may give inappropriate dietary advice. This may be a reason for immigrants to quit dietetic treatment. Compared to other frequent diagnoses in this study, patients with overweight, binge eating disorder, or multiple diagnoses were strongly associated with using more consultations per treatment. This could be explained by the complexity of these health problems and underlying issues. No significant relation between consultation sessions and communication problems or intellectual disability was found. Possibly, a positive relation could be found in other health care settings, as this study sample consisted of dietitians working in general solo practices not specialized in treating patients with communication problems or intellectual disability.

A strength of the study is the use of routine registration as facilitated by the LiPZ software. This meant the data was continuously collected with the software program that dietitians use for regular practice administration, and additional questions were completed by the RD during the consultation or shortly afterwards. Therefore, there is little risk for recall bias. Furthermore, minimal inaccuracies are expected regarding the outcome variable as the registration was based on reimbursement claims. Aside from the advantages, some limitations of the study should be taken into account when interpreting the results. There is a possibility that the participants working in solo practices constitute a subgroup of all Dutch dietitians working in private practice. However, there is no national information available about the number of dietitians working in private practices in the Netherlands. Additionally, the number of participating dietitians in this study was too small to study more therapist-related factors in order to explain inter-practitioner variation (n=27). Therefore more research is necessary with a larger number of practitioners. In the Dutch situation dietetic treatment is reimbursed by insurance companies for up to a maximum of four hours per calendar year. Therefore, the effect of reimbursement on consultation sessions was not taken into account. Probably reimbursement will play a large role in dietetic healthcare use in other countries, as in many countries dietetic treatment is not or only partly reimbursed by insurance companies [34-36]. Therefore the patient population of this study may differ from the patient population in other countries – e.g. on social economic status or motivation. As costs have a major impact on patient retention, it can be hypothesized that the patient’s motivation increases when dietetic treatment is not reimbursed. More international research on these topics will increase the transparency of dietetic treatment in a more universal perspective.

## Conclusion

In conclusion it was found that there is considerable variation in number of consultations per dietetic treatment which is due to dietitians. Some of this inter-practitioner variation was reduced after adjusting for case-mix. Further research is necessary to study the relation between inter-practitioner variation and the effectiveness and quality of dietetic treatment.

## Competing interests

This study has no competing interests. This work was performed by NIVEL, the Netherlands Institute for Health Services Research. A steering committee has been established in order to assist the NIVEL in executing the LiPZ project. Current members of the committee are representatives of the Dutch association of Dietitians (NVD), the Royal Dutch Society for Physical Therapy (KNGF), the association of Cesar and Mensendieck therapists (VvOCM), and the Dutch Health Insurers association (ZN). In addition, the committee can receive advice from the college of Dutch Health Insurers (CvZ), and the Dutch Healthcare Authority (NZa).

## Authors’ contributions

JT, IS, CL, CV were involved in het conception of the research question. JT and PS were involved in analysing the data. All authors contributed to the interpretation of the data. JT drafted the manuscript, which was reviewed and approved by all authors.

## Pre-publication history

The pre-publication history for this paper can be accessed here:

http://www.biomedcentral.com/1472-6963/12/317/prepub

## Supplementary Material

Additional file 1 Organization of dietetics in The Netherlands. Click here for file
