# Supplementary material for: Factors associated with the number of consultations per dietetic treatment: an observational study
Source: BMC Health Serv Res. 2012 Sep 14;12:317. doi: 10.1186/1472-6963-12-317 (PMC3502485; doi:10.1186/1472-6963-12-317)
Supplement: Additional file 1 — Organization of dietetics in The Netherlands. [file 1472-6963-12-317-S1.pdf]

Additional file 1: Organization of dietetics in The Netherlands

In 2009, about 45% of all Dutch registered dietitians (RD) worked in primary health care and 60% of them worked in private practice [1].

All Dutch citizens have to buy standardized individual primary health insurance coverage from a private insurer, and the government of the Netherlands subsidies so that primary health insurance is affordable for everyone.

Since January 1st 2005, dietetic treatment was reimbursed by the standardized primary health insurance coverage for up to a maximum of four hours per calendar year (January 1st - December 31st), under the condition that the patient had a medical indication and was referred by a physician. This reimbursement includes the direct treatment time, i.e. the total time of the consultation with the patient, and the indirect treatment time, i.e. the time the dietitian needs to administer and prepare the patient's consultation. For a higher premium a patient can buy extra coverage, for extra treatment time or unlimited reimbursement of dietetic treatment [2]. All reimbursement also covers treatment for overweight and obesity.

The Dutch situation has changed in 2011. Since August 2011 dietetic health care is accessible without a referral from a physician. From January 2012 dietetic health care will only be reimbursed by the standardized individual primary health insurance coverage for up to a maximum of four hours a year, under the condition that the patient receives integrated care and is diagnosed with diabetes mellitus, chronic obstructive pulmonary disease or has a cardiovascular risk.

1. The Dutch Dietetic Association (NVD): **Statistics of the Dutch dietetic association**. In *Dutch: statistieken NVD*. http://www.nvdietist.nl/content.asp?kid=10529458.

2. The Ministry of Health, Welfare and Sport: *Primary health care in The Netherlands*. The Hague: Report; 2005.
